# The relationship between metabolic syndrome and survival of patients with endometrial cancer: a meta-analysis

**DOI:** 10.3389/fonc.2024.1484109

**Published:** 2024-10-21

**Authors:** Feng Deng, Yi Chen, Ying Wu, Yawen Tang, Wangjun Yi

**Affiliations:** Department of Gynecology, The Third Hospital of Changsha, Changsha, Hunan, China

**Keywords:** metabolic syndrome, endometrial cancer, survival, prognosis, meta-

## Abstract

**Background:**

Metabolic syndrome (MetS) is associated with a high risk of endometrial cancer (EC). However, its impact on EC progression remains unclear. This meta-analysis examined the association between MetS and survival outcomes in EC patients.

**Methods:**

A comprehensive search of PubMed, EMBASE, and Web of Science databases up to May 22, 2024, was conducted. Two independent reviewers performed study selection, data extraction, and quality assessment. Hazard ratios (HRs) and 95% confidence intervals (CIs) were calculated using a random effects model.

**Results:**

Nine studies comprising 13,579 endometrial cancer (EC) patients were included. Among these, 2,896 patients (21.3%) had MetS at the time of enrollment. The follow-up durations ranged from 3.4 to 14.2 years. The results showed that EC patients with MetS at baseline demonstrated significantly poorer overall survival (HR = 1.57, 95% CI = 1.19–2.07, *p* = 0.002; I^2^ = 25%) and progression-free survival (HR = 1.33, 95% CI = 1.08–1.63, *p* = 0.007; I^2^ = 16%). A similar association was observed for cancer-specific survival (HR = 1.26, 95% CI = 1.10–1.44, *p* = 0.001; I^2^ = 0%). Subgroup analyses based on study characteristics showed consistent results across studies conducted in countries with different follow-up durations.

**Conclusion:**

This meta-analysis suggests that MetS is associated with poor survival outcomes in EC patients. Further prospective studies are required to validate our findings.

**Systematic review registration:**

PROSPERO, identifier CRD42024561654.

## Introduction

Endometrial cancer (EC) is a significant global health burden and the most common gynecological malignancy in developed countries (1). Its incidence has been rising steadily, primarily due to increasing obesity rates and aging populations (2, 3). EC typically manifests in postmenopausal women and is notably associated with hormonal imbalances, particularly estrogen dominance (4). The prognosis varies widely depending on the disease stage at diagnosis, with early-stage tumors generally having favorable outcomes due to effective surgical interventions and adjuvant therapies (5–8). However, advanced stages pose considerable challenges in management and are often associated with poor survival rates, despite aggressive treatment approaches (9).

Metabolic syndrome (MetS) comprises a cluster of related risk factors including central obesity, hypertension, dyslipidemia, and insulin resistance (10). This syndrome has garnered attention not only for its role in cardiovascular disease, but also for its potential impact on cancer development and progression (11). Epidemiological studies have indicated that individuals with MetS are at increased risk of several cancers (12), including EC (13, 14). The underlying mechanisms linking MetS to cancer involve chronic inflammation, hyperinsulinemia, and altered hormone metabolism, which collectively create a tumor-promoting microenvironment (15, 16).

The association between MetS and cancer outcomes, particularly EC, remains an area of active investigation (17). Although MetS has been implicated in the pathogenesis of EC through its influence on hormonal profiles and chronic inflammation (18), its specific impact on survival outcomes of patients with EC is less well defined. Understanding this relationship is crucial as it may inform strategies for risk stratification, treatment optimization, and patient counseling. However, pilot studies on the effects of MetS on survival outcomes in women with EC have yielded inconsistent results (19–27). To address this knowledge gap, this meta-analysis systematically evaluated existing evidence on the association between MetS and survival outcomes in patients with EC. Because of this knowledge gap, this meta-analysis aimed to systematically evaluate existing evidence regarding the association between MetS and survival outcomes in patients with EC.

## Methods

This meta-analysis adhered to the Preferred Reporting Items for Systematic Reviews and Meta-Analyses (PRISMA 2020) guidelines (28, 29) and the Cochrane Handbook for Systematic Reviews and Meta-Analyses (30) throughout its design, data collection, statistical analysis, and interpretation of the results. The meta-analysis protocol was registered in PROSPERO (registration number CRD42024561654).

### Data sources and search strategy

A comprehensive literature search was performed using PubMed, EMBASE, and Web of Sciences to identify relevant cohort studies published from database inception to May 22, 2024. The search strategy included the combined terms of (1) “metabolic syndrome” OR “insulin resistance syndrome” OR “syndrome X”; (2) “endometrial” OR “uterine” OR “myometrial”; (3) “cancer” OR “tumor” OR “neoplasm” OR “carcinoma” OR “malignancy”; and (4) “survival” OR “death” OR “mortality” OR “prognosis” OR “recurrence” OR “recurrent” OR “progression” or “overall survival” OR “progression-free survival” OR “prospective” OR “retrospective” OR “followed” OR “follow-up” OR “longitudinal” OR “risk” OR “incidence.” Only studies published in English as full-length articles in peer-reviewed journals were included. In addition, the reference lists of the identified articles and relevant reviews were screened to ensure comprehensive coverage.

### Study selection

Studies were included if they met the following criteria and were designed according to the PICOS model:

P (patients): women with a confirmed diagnosis of EC without cancer stage or treatment limitations.

I (exposure): patients with MetS at baseline who were diagnosed according to the criteria used in the original studies.

C (comparison): Patients without MetS at baseline.

O (outcome): reported at least one of the following outcomes compared between patients with and without MetS at baseline: overall survival (OS), progression-free survival (PFS), or cancer-specific survival (CSS). OS was defined as the time from enrollment to death from any cause. PFS was defined as the interval between enrollment and first EC recurrence or progression. CSS was defined as the time from enrollment to death, specifically from EC.

S (study design): longitudinal studies, including cohort studies, nested case-control studies, and *post-hoc* analyses of clinical trials.

The exclusion criteria were reviews, editorials, meta-analyses, preclinical studies, cross-sectional studies, studies involving patients with cancers other than EC, and studies that did not report survival outcomes. For studies with overlapping patient populations, the study with the largest sample size was chosen for meta-analysis.

### Quality evaluation and data extraction

Two authors independently performed literature search, study identification, quality evaluation, and data collection. Disagreements were resolved through discussion with the corresponding author to reach a consensus. Study quality was assessed using the Newcastle-Ottawa Scale (NOS) (31), which evaluates studies based on the selection of the study population, comparability between groups, and measurement of exposure. NOS scores ranged from 0 to 9, with higher scores indicating better study quality. A score of 7-9 was considered high quality. The data extracted from each study included study details (authors, year, design, country), patient characteristics (sample size, age, histological type of EC, tumor stage, main treatments), MetS diagnostic criteria, number of patients with MetS at enrollment, follow-up duration, reported outcomes, and variables adjusted for, to evaluate the association between MetS and EC survival outcomes.

### Statistical analysis

The association between MetS and survival outcomes in EC was summarized using hazard ratios (HRs) and 95% confidence intervals (CIs). HRs and standard errors (SEs) were calculated from 95% CIs or p-values, and logarithmic transformation was applied to stabilize and normalize variance. Study heterogeneity was assessed using the Cochrane Q test and I² statistics, with I² > 50% indicating significant heterogeneity (32). Given the clinical variability among the studies (e.g., patient characteristics, treatments, and MetS definitions), a random-effects model was used to account for between-study heterogeneity (30). Sensitivity analyses were performed by sequentially omitting each study in order to test the robustness of the results. A predefined subgroup analysis was performed to evaluate how study characteristics, such as country, tumor stage, and follow-up duration, affected the meta-analysis outcomes, using medians as cutoffs for subgroup definitions. Publication bias was initially assessed using funnel plots and visual inspection of symmetry (33) followed by Egger’s regression test (33). Statistical analyses were performed using RevMan (Version 5.1; Cochrane Collaboration, Oxford, UK) and Stata (version 12.0; Stata Corporation, College Station, TX, USA), with a two-sided p-value < 0.05 considered statistically significant.

## Results

### Database search and study inclusion

**Figure 1:**
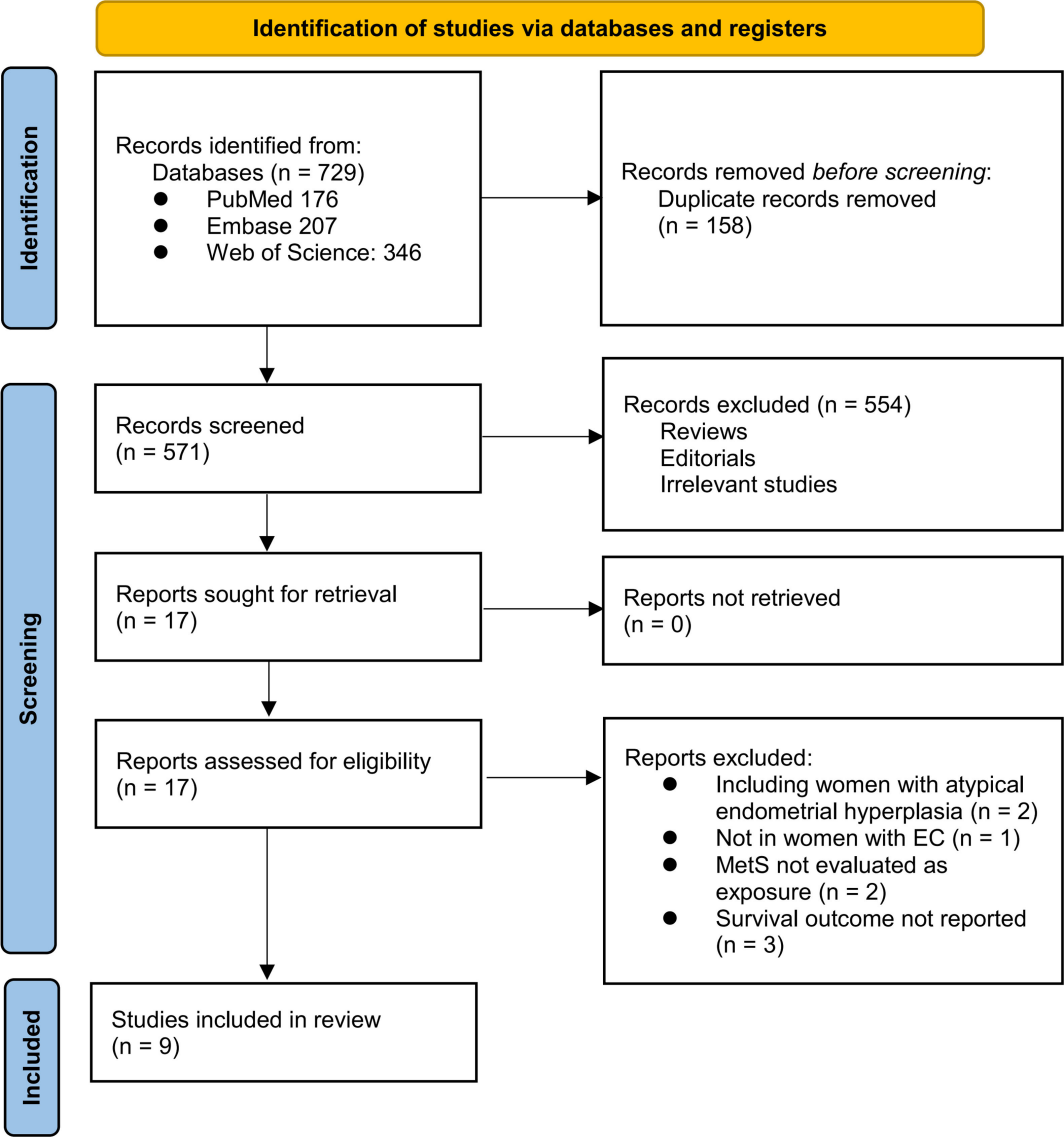
The flowchart shows the database search process and study inclusion.

The study inclusion process is illustrated in **Figure 1**. Initially, 729 potentially relevant records were retrieved from the three databases, of which 158 were removed because of duplication. After screening the titles and abstracts, 554 studies were excluded primarily because they were not pertinent to the meta-analysis. Two independent authors reviewed the full texts of the remaining 17 records and excluded eight additional studies for the reasons detailed in **Figure 1**. Ultimately, nine longitudinal observational studies were deemed suitable for quantitative analysis (19–27).

### Characteristics of the included studies

**Table 1** summarizes the characteristics of the included studies. The meta-analysis included eight retrospective cohort studies (19, 20, 22–27) and one nested case-control study (21). These studies, published between 2015 and 2024, were conducted in China, the United States, Canada, Malaysia, and Germany. A total of 13,579 women with EC were included, with mean ages ranging from 52.5 to 74.8 years across the studies. Histologically, endometrioid EC accounted for 85.4% of the included patients. Surgical resection was the primary treatment in seven included studies (21, 23). Comprehensive treatment involving surgery, chemotherapy, radiotherapy, or hormone therapy was used in one study (21). In contrast, another study did not mention the primary anticancer treatment (23). The diagnostic criteria included National Cholesterol Education Program Adult Treatment Panel III criteria (20, 21), International Diabetes Foundation criteria (19, 22, 23, 25), Chinese Diabetes Society criteria (24, 26), and clinically diagnosed MetS based on the presence of its components (27). Accordingly, 2896 (21.3%) of the included patients had MetS at enrollment. The mean follow-up duration was 3.4 to 14.2 years. The OS was reported in seven studies (19, 21–25, 27), PFS in six studies (21, 23–27), and CSS in two studies (20, 21). Multivariate regression analysis was performed in eight studies when the association between MetS and survival outcomes of EC was analyzed (19–22, 24–27). In contrast, a univariate regression analysis was performed in another study (23). The NOS scores for the included studies ranged from six to eight stars, indicating an overall moderate to good study quality (**Table 2**).

**Table 1:** Study characteristics.

Study	Study design	Location	Sample size	Mean age (years)	Histology	FIGO Stage	Main treatment	Definition of MetS	No. of patients with MetS	Median follow-up durations (years)	Outcomes	Variables adjusted
Ni 2015	RC	China	385	55	Endometrioid (100%)	I-IV	Surgery with or without adjuvant therapy	IDF	129	6	OS	Age, tumor grade, stage, size, vascular invasion, and lymphatic metastasis
Jin 2020	RC	USA	10090	74.8	Endometrioid (83.9%)	I-IVa	Surgery with or without adjuvant therapy	NCEP-ATP III	1612	6	CSS	Age, race, income, year of diagnosis, histopathology, and adjuvant treatment
Kokts-Porietis 2020	NCC	Canada	540	59.1	Endometrioid (81.5%)	I-III	Comprehensive (surgery, chemotherapy, radiotherapy, or hormone therapy)	NCEP-ATP III	325	14.2	PFS, OS, and CSS	Age, BMI, tumor grade, stage, and primary anticancer treatment
Shou 2020	RC	China	139	56	Non-endometrioid	I-IV	Surgery with or without adjuvant therapy	IDF	41	8.3	OS	Age, postmenopausal, tumor stage, and adjuvant therapy
Yang 2021	RC	China	506	55.8	Endometrioid (86.2%)	I-IV	Surgery with or without adjuvant therapy	CDS	153	4.2	PFS and OS	Age, tumor histotype, grade, and stage
Shafiee 2021	RC	Malaysia	119	55.3	Endometrioid (86%)	NR	NR	IDF	65	5	PFS and OS	None
Wang 2022	RC	China	998	NR	Endometrioid (100%)	I-II	Surgery with or without adjuvant therapy	CDS	339	3.4	PFS	Age, tumor grade, family history, and LVSI
Chen 2022	RC	China	387	52.5	Endometrioid (100%)	I	Surgery with or without adjuvant therapy	IDF	194	3.4	PFS and OS	Age, menopause, tumor grade, size, LVSI, deep myometrial invasion, surgery type, and adjuvant treatment
Shehaj 2024	RC	Germany	415	64.4	Endometrioid (92.3%)	I-IV	Surgery with or without adjuvant therapy	Clinically diagnosed	38	3.6	PFS and OS	Age, BMI, ECOG score, histotype, tumor grade, stage, LVSI, surgery type, and adjuvant treatment

MetS, metabolic syndrome; RC, retrospective cohort; NCC, nested case-control; NR, not reported; IDF, International Diabetes Foundation; NCEP-ATP III, National Cholesterol Education Program Adult Treatment Panel III; CDS, Chinese Diabetes Society; OS, overall survival; CSS, cancer-specific survival; PFS, progression-free survival; BMI, body mass index; LVSI, lymphovascular space invasion; ECOG, Eastern Cooperative Oncology Group; FIGO, International Federation of Gynecology and Obstetrics

**Table 2:** Study quality evaluation via Newcastle-Ottawa Scale.

Cohort Study	Representativeness of the exposed cohort	Selection of the non-exposed cohort	Ascertainment of exposure	Outcome not present at baseline	Control for age and sex	Control for other confounding factors	Assessment of outcome	Enough long follow-up duration	Adequacy of follow-up of cohorts	Total
Ni 2015	0	1	1	1	1	1	1	1	1	8
Jin 2020	0	1	1	1	1	1	1	1	1	8
Kokts-Porietis 2020	0	1	1	1	1	1	1	1	1	8
Shou 2020	0	1	1	1	1	1	1	1	1	8
Yang 2021	0	1	1	1	1	1	1	0	1	7
Shafiee 2021	0	1	1	1	0	0	1	1	1	6
Wang 2022	0	1	1	1	1	1	1	0	1	7
Chen 2022	0	1	1	1	1	1	1	0	1	7
Shehaj 2024	0	1	1	1	1	1	1	0	1	7

### Association between MetS and OS

**Figure 2:**
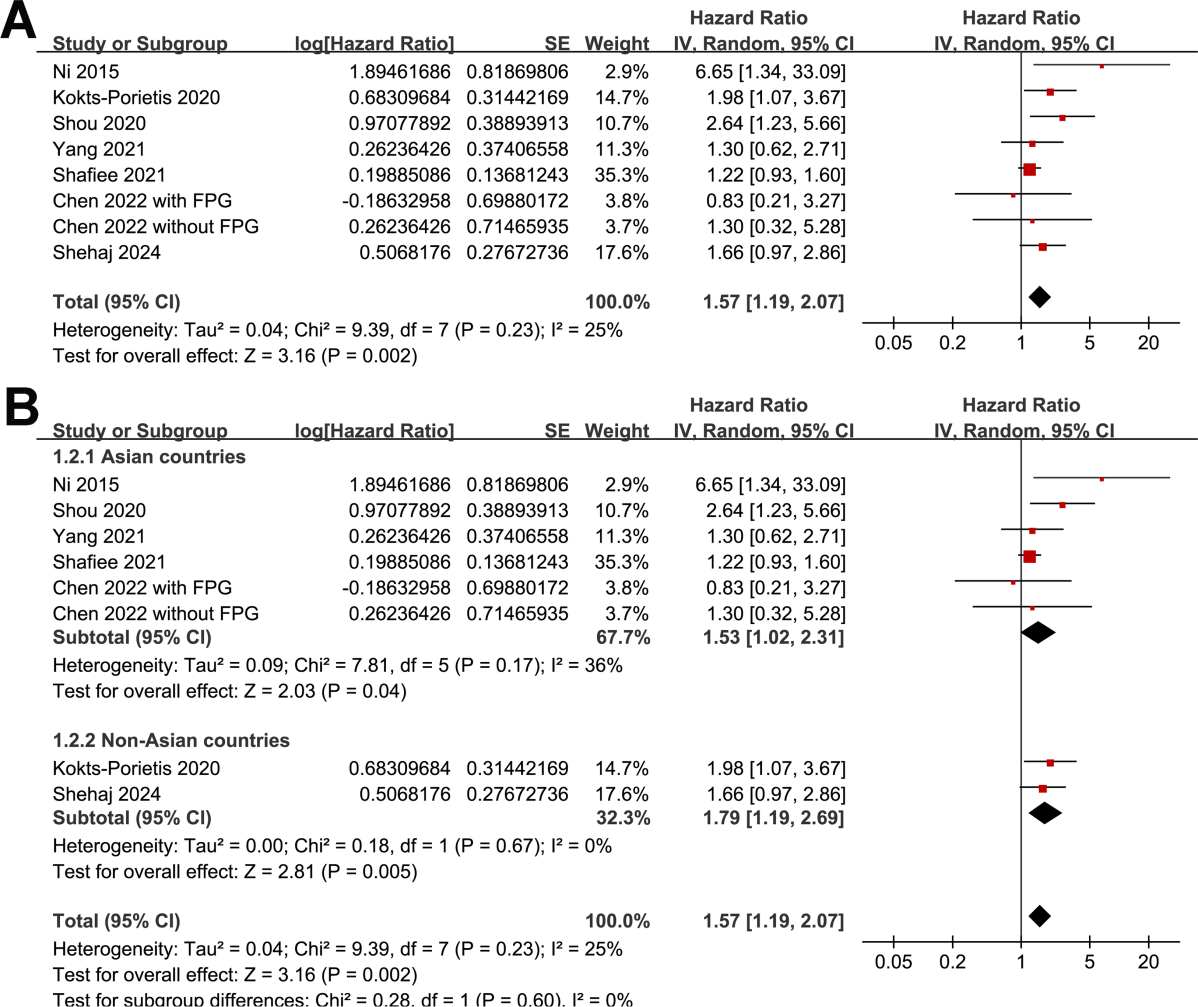
Forest plots for the meta-analysis of the association between MetS and OS in patients with EC: **(A)**, forest plots for the overall meta-analysis; **(B)**, forest plots for the subgroup analysis according to the study country.

**Figure 3:**
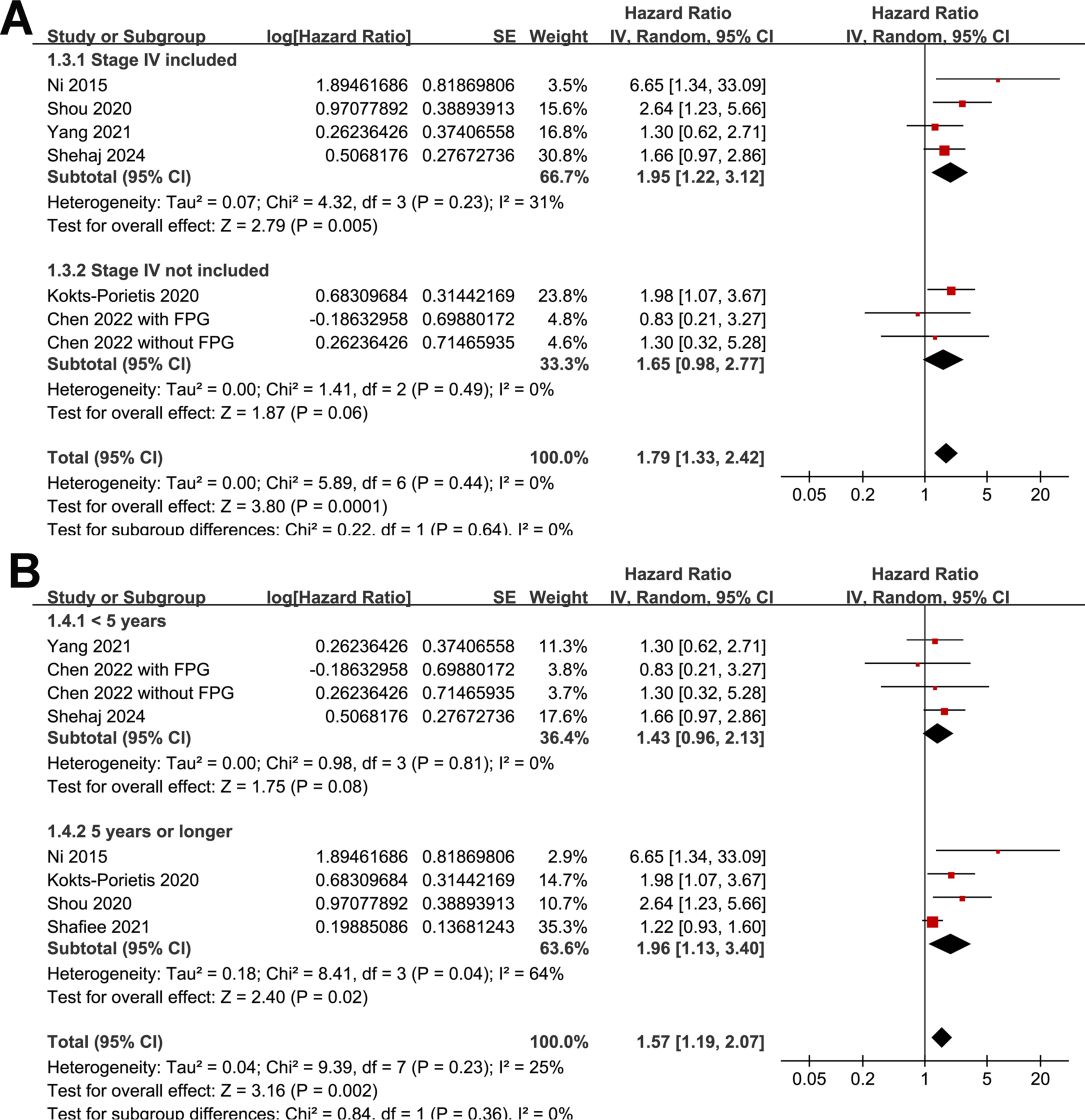
Forest plots for subgroup analyses of the association between MetS and OS of patients with EC; **(A)**, forest plots for subgroup analysis according to whether patients with stage IV EC were included; **(B)**, forest plots for subgroup analysis according to follow-up duration.

Because one study separately reported the outcome of MetS patients with and without impaired fasting plasma glucose (25), these datasets were independently included in the meta-analysis. The pooled results of eight datasets from seven studies (19, 21–25, 27) revealed that EC patients with MetS at enrollment had poorer OS than those without MetS (HR = 1.57, 95% CI = 1.19–2.07, *p* = 0.002; I^2^ = 25%; **Figure 2A**). The sensitivity analysis, by omitting one study at a time, did not significantly change the results (HR: 1.41–1.79, *p* < 0.05). Further subgroup analyses showed similar results in studies from Asian and non-Asian countries (p for subgroup difference = 0.60; **Figure 2B**), with and without patients with stage IV EC (p for subgroup difference = 0.64; **Figure 3A**), and in studies with a follow-up duration of ≤ or ≥ 5 years (*p* for subgroup difference = 0.36; **Figure 3B**).

### Association between MetS and PFS

**Figure 4:**
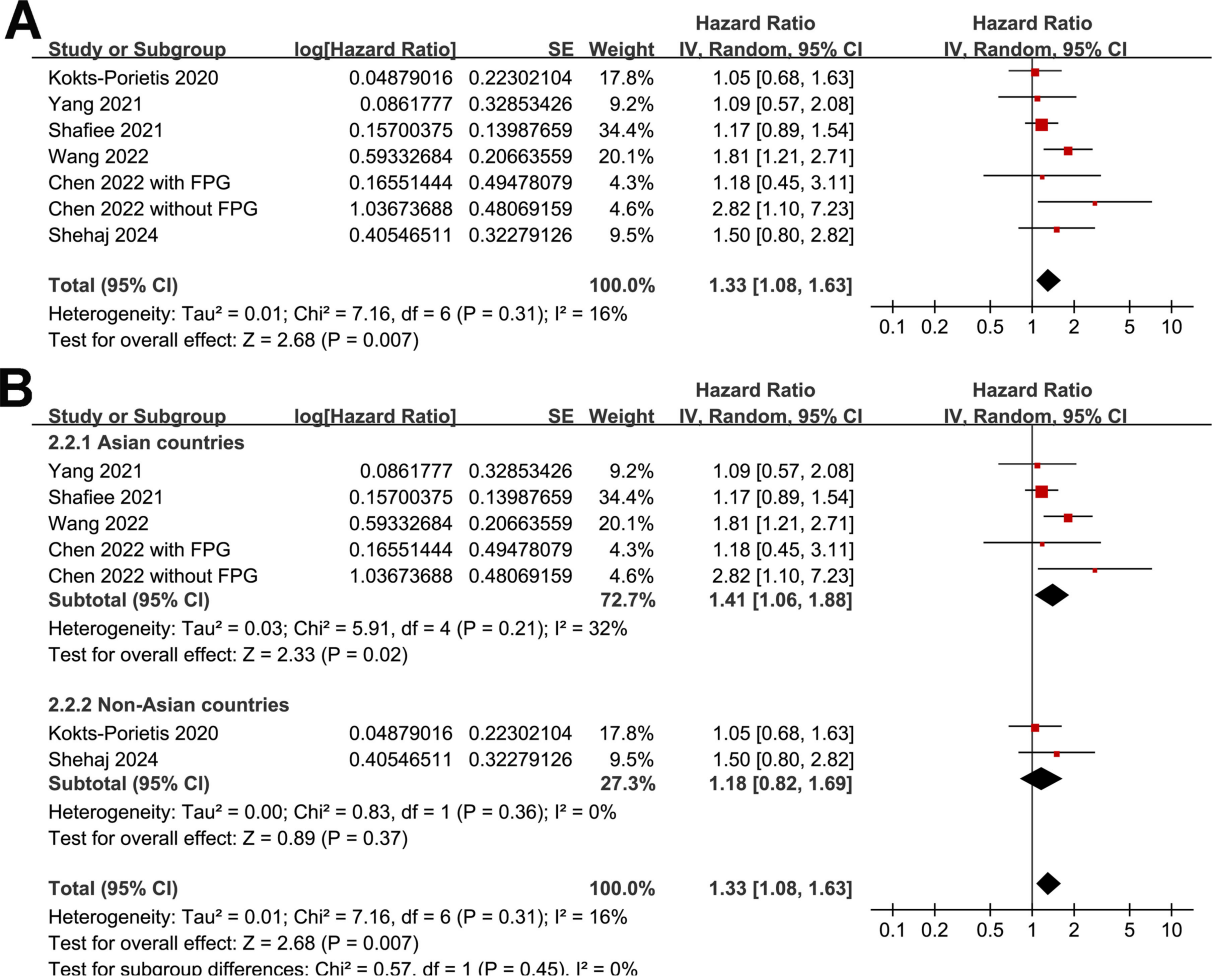
Forest plots for the meta-analysis of the association between MetS and PFS in patients with EC: **(A)**, forest plots for the overall meta-analysis; **(B)**, forest plots for subgroup analysis according to the study country.

**Figure 5:**
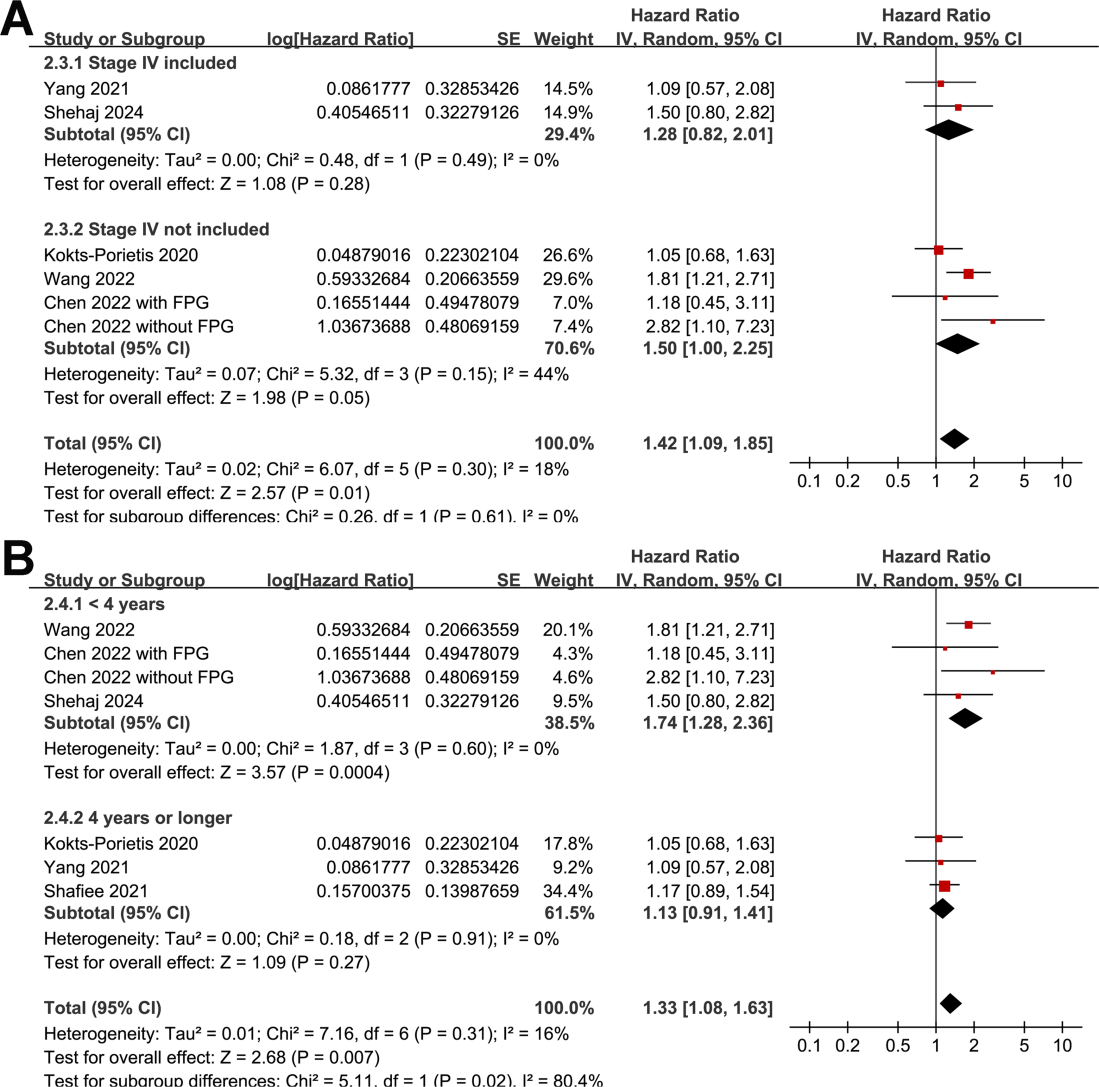
Forest plots for the subgroup analyses of the association between MetS and PFS of patients with EC; **(A)**, forest plots for the subgroup analysis according to whether patients with stage IV EC were included; **(B)**, forest plots for the subgroup analysis according to the follow-up duration.

A meta-analysis of seven datasets from six studies (21, 23–27) indicated poor PFS in patients with EC who had MetS at enrollment compared with those without MetS (HR = 1.33, 95% CI = 1.08–1.63, *p* = 0.007; I^2^ = 16%; **Figure 4A**). Sensitivity analysis, excluding one dataset at a time, showed similar results (HR: 1.21–1.42, p < 0.05). Further subgroup analyses showed similar results in studies from Asian and non-Asian countries (*P* for subgroup difference = 0.45; **Figure 4B**) and studies with and without patients with stage IV EC (*P* for subgroup difference = 0.61; **Figure 5A**). Interestingly, a subgroup analysis suggested that the association between MetS and PFS in women with EC was stronger in studies with a follow-up duration of < 4 years than in those with a follow-up duration of ≥ 4 years (*p* for subgroup difference = 0.02; **Figure 5B**).

### Association between MetS and CSS

**Figure 6:**
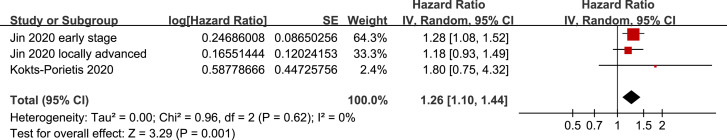
Forest plots for the meta-analysis of the association between MetS and CSS in patients with EC.

Because one study separately reported the outcome of CSS in patients with early-stage and locally advanced EC (20), these datasets were independently included in the meta-analysis. The pooled results of three datasets from two studies (20, 21) suggested that MetS was also associated with poor CSS in women with EC (HR = 1.26, 95% CI = 1.10–1.44, *p* = 0.001; I^2^ = 0%; **Figure 6**).

### Publication bias

**Figure 7:**
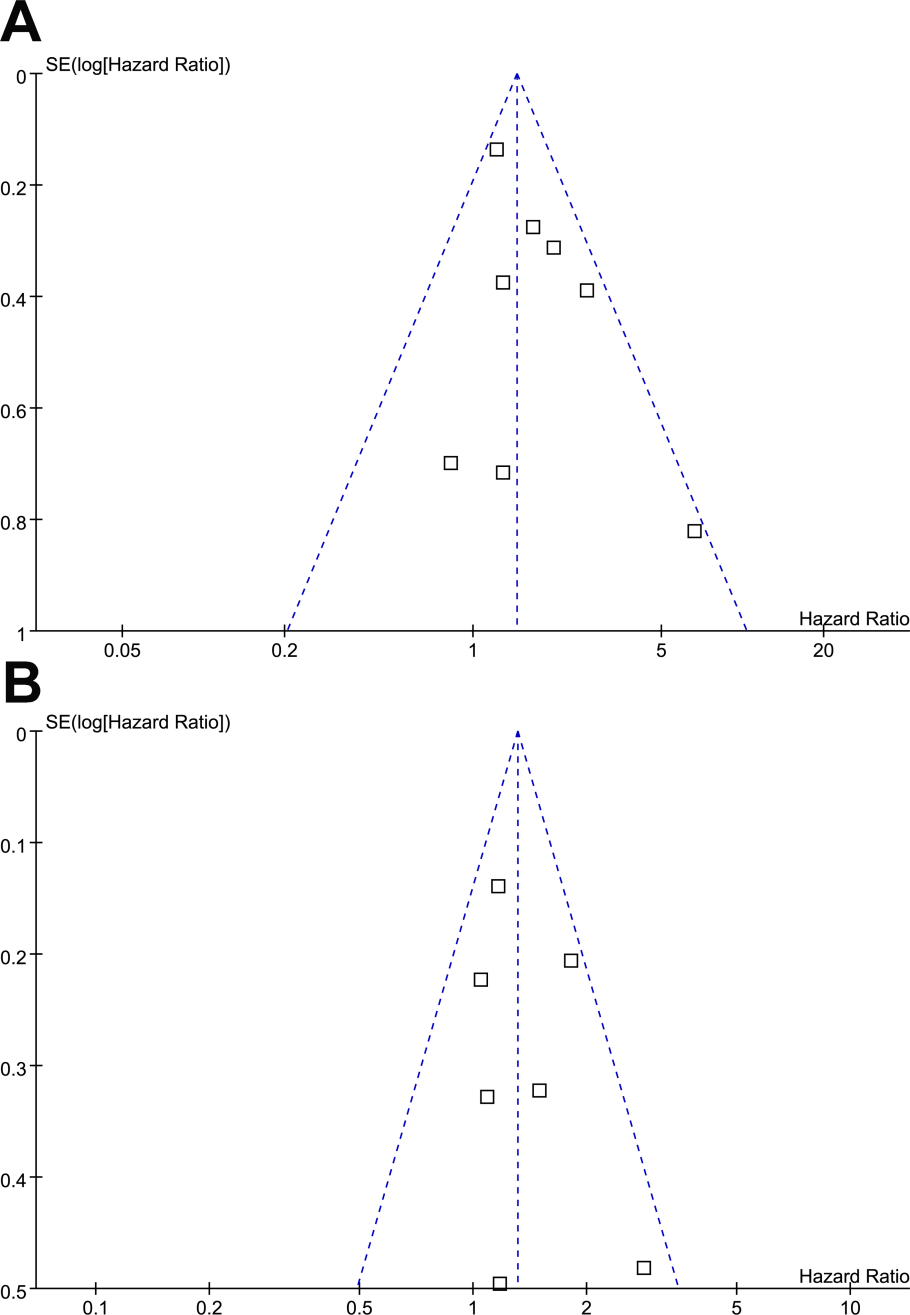
Funnel plots for the meta-analysis of the associations of MetS with OS and PFS in patients with EC: **(A)**, funnel plots for the outcome of OS; **(B)**, funnel plots for the outcome of PFS.

Funnel plots for MetS associations with OS and PFS in EC patients are shown in **Figures 7A, B**. The plots appeared symmetrical, suggesting minimal publication bias. Egger’s tests further confirmed low publication bias for OS and PFS (p = 0.59 and 0.33, respectively). Assessment of publication bias for CSS was impossible because of the limited number of datasets (three).

## Discussion

In this study, we systematically synthesized data from nine longitudinal studies, and pooled analysis revealed a significant association between MetS and adverse survival outcomes in patients with EC, including poorer OS, PFS, and CSS. Specifically, EC patients with MetS at baseline exhibited a 57% higher mortality risk than those without MetS, highlighting MetS as a potential prognostic factor in EC management. The subgroup analyses conducted in this meta-analysis provided further insights into the consistency of the associations across different study populations. Subgroups based on geographical location, disease stage, and follow-up duration consistently showed that MetS adversely affected EC survival outcomes, irrespective of these variables. Sensitivity analyses confirmed the robustness of the findings as the association between MetS and EC survival outcomes remained significant when individual studies were omitted. These analyses underscored the reliability and validity of the observed associations.

The association between MetS and EC survival outcomes can be attributed to several underlying molecular mechanisms. MetS components, such as central obesity, insulin resistance, dyslipidemia, and hypertension, create a milieu conducive to cancer progression (34). For instance, insulin resistance leads to hyperinsulinemia and increased bioavailability of insulin-like growth factors (IGFs), which promote cell proliferation and inhibit apoptosis in cancer cells (35, 36). Moreover, adipose tissue in MetS secretes pro-inflammatory cytokines and adipokines, fostering a chronic inflammatory state that supports tumor growth and metastasis (37, 38). Collectively, these mechanisms contribute to a more aggressive tumor phenotype and reduced treatment response in patients with EC and MetS. Furthermore, prior research has demonstrated that MetS significantly affects postoperative complications among EC patients and may hinder the achievement of disease-free status in some cases (39). Similarly, recent studies have highlighted that the presence of MetS before surgery can predict the likelihood of myometrial invasion in EC (40). These insights contribute to our understanding of the potential contribution of MetS to poor survival outcomes in patients with EC. In addition, we acknowledge that the observed association between MetS and poorer OS in patients with EC could be partly due to surgical undertreatment or non-adherence to clinical guidelines, potentially driven by the complexity of managing multiple comorbidities. Furthermore, it is plausible that other comorbidities commonly associated with MetS, such as cardiovascular disease and diabetes, may independently affect OS by reducing life expectancy. However, it is important to note that there is no direct evidence in the literature that confirms these hypotheses in the context of EC. Future studies should explore the extent to which these factors may contribute to disparities in survival among patients with EC and MetS.

To our knowledge, this is the first meta-analysis to systematically evaluate the impact of MetS on the survival outcomes of patients with EC. Despite the strengths of this meta-analysis, including its comprehensive search strategy and rigorous methodology adhering to the PRISMA guidelines, several limitations must be acknowledged. First, most of the included studies were retrospective cohort studies, susceptible to selection and recall biases. In addition, variability in study design, definitions of MetS, and treatment modalities across studies also introduced heterogeneity that may have influenced the pooled effect estimates. Additionally, although efforts were made to explore the sources of heterogeneity through subgroup analyses, residual confounding factors and unmeasured variables could not be fully excluded. Moreover, owing to the limited number of available datasets, we could not determine the influence of cancer histology and primary treatment on the association between MetS and EC survival. Further studies are required to confirm these findings. Finally, because this was a meta-analysis of observational studies, a causative relationship between MetS and poor prognosis of EC could not be derived based on the findings.

Although large-scale prospective studies are needed to validate our findings, their clinical implications may be significant for EC management. Routine assessment of MetS components may be integrated into the clinical evaluation of patients with EC to identify those at a higher risk of adverse outcomes. More importantly, it is essential to evaluate whether the early recognition and management of MetS through lifestyle modifications and pharmacological interventions can improve metabolic parameters and subsequently enhance treatment efficacy and patient survival. If confirmed, clinicians should consider incorporating MetS management strategies into personalized treatment plans for EC patients to optimize outcomes.

## Conclusion

In conclusion, this meta-analysis provides pilot evidence that MetS is associated with poor survival outcomes in patients with EC. These findings underscore the potential importance of addressing metabolic health in EC management strategies and highlight the potential impact of MetS on treatment response and overall prognosis. Future studies should focus on elucidating the specific molecular pathways linking MetS to EC progression, conducting prospective studies to validate these findings, and exploring targeted therapeutic interventions to mitigate the adverse effects of MetS on EC outcomes.

## Data Availability

The original contributions presented in the study are included in the article/supplementary material. Further inquiries can be directed to the corresponding author.

## References

[1] Siegel RL Giaquinto AN Jemal A . Cancer statistics, 2024. CA Cancer J Clin. (2024) 74:12–49. doi: 10.3322/caac.21820 38230766

[2] Zhang S Gong TT Liu FH Jiang YT Sun H Ma XX . Global, regional, and national burden of endometrial cancer, 1990-2017: results from the global burden of disease study, 2017. Front Oncol. (2019) 9:1440. doi: 10.3389/fonc.2019.01440 31921687 PMC6930915

[3] Feng J Lin R Li H Wang J He H . Global and regional trends in the incidence and mortality burden of endometrial cancer, 1990-2019: Updated results from the Global Burden of Disease Study, 2019. Chin Med J (Engl). (2024) 137:294–302. doi: 10.1097/CM9.0000000000002841 37874032 PMC10836881

[4] Makker V MacKay H Ray-Coquard I Levine DA Westin SN Aoki D . Endometrial cancer. Nat Rev Dis Primers. (2021) 7:88. doi: 10.1038/s41572-021-00324-8 34887451 PMC9421940

[5] Crosbie EJ Kitson SJ McAlpine JN Mukhopadhyay A Powell ME Singh N . Endometrial cancer. Lancet. (2022) 399:1412–28. doi: 10.1016/S0140-6736(22)00323-3 35397864

[6] Lu KH Broaddus RR . Endometrial cancer. N Engl J Med. (2020) 383:2053–64. doi: 10.1056/NEJMra1514010 33207095

[7] Restaino S Paglietti C Arcieri M Biasioli A Della Martina M Mariuzzi L . Management of patients diagnosed with endometrial cancer: comparison of guidelines. Cancers (Basel). (2023) 15(4):1091. doi: 10.3390/cancers15041091 36831434 PMC9954548

[8] Corrado G Ciccarone F Cosentino F Legge F Rosati A Arcieri M . Role of minimally invasive surgery versus open approach in patients with early-stage uterine carcinosarcomas: a retrospective multicentric study. J Cancer Res Clin Oncol. (2021) 147:845–52. doi: 10.1007/s00432-020-03372-x PMC7873090 32880752

[9] Tronconi F Nero C Giudice E Salutari V Musacchio L Ricci C . Advanced and recurrent endometrial cancer: State of the art and future perspectives. Crit Rev Oncol Hematol. (2022) 180:103851. doi: 10.1016/j.critrevonc.2022.103851 36257537

[10] Fahed G Aoun L Bou Zerdan M Allam S Bouferraa Y Assi HI . Metabolic syndrome: updates on pathophysiology and management in 2021. Int J Mol Sci. (2022) 23(2):786. doi: 10.3390/ijms23020786 35054972 PMC8775991

[11] Belladelli F Montorsi F Martini A . Metabolic syndrome, obesity and cancer risk. Curr Opin Urol. (2022) 32:594–7. doi: 10.1097/MOU.0000000000001041 36081396

[12] Esposito K Chiodini P Colao A Lenzi A Giugliano D . Metabolic syndrome and risk of cancer: a systematic review and meta-analysis. Diabetes Care. (2012) 35:2402–11. doi: 10.2337/dc12-0336 PMC3476894 23093685

[13] Wang L Du ZH Qiao JM Gao S . Association between metabolic syndrome and endometrial cancer risk: a systematic review and meta-analysis of observational studies. Aging (Albany NY). (2020) 12:9825–39. doi: 10.18632/aging.103247 PMC7288955 32439832

[14] Esposito K Chiodini P Capuano A Bellastella G Maiorino MI Giugliano D . Metabolic syndrome and endometrial cancer: a meta-analysis. Endocrine. (2014) 45:28–36. doi: 10.1007/s12020-013-9973-3 23640372

[15] Mili N Paschou SA Goulis DG Dimopoulos MA Lambrinoudaki I Psaltopoulou T . Obesity, metabolic syndrome, and cancer: pathophysiological and therapeutic associations. Endocrine. (2021) 74:478–97. doi: 10.1007/s12020-021-02884-x 34625915

[16] Yang X Wang J . The role of metabolic syndrome in endometrial cancer: A review. Front Oncol. (2019) 9:744. doi: 10.3389/fonc.2019.00744 31440472 PMC6694738

[17] Marin AG Filipescu A Vladareanu R Petca A . Metabolic syndrome and survival outcomes in endometrial cancer. Cureus. (2024) 16:e60324. doi: 10.7759/cureus.60324 38883006 PMC11177328

[18] Perez-Martin AR Castro-Eguiluz D Cetina-Perez L Velasco-Torres Y Bahena-Gonzalez A Montes-Servin E . Impact of metabolic syndrome on the risk of endometrial cancer and the role of lifestyle in prevention. Bosn J Basic Med Sci. (2022) 22:499–510. doi: 10.17305/bjbms.2021.6963 35276057 PMC9392984

[19] Ni J Zhu T Zhao L Che F Chen Y Shou H . Metabolic syndrome is an independent prognostic factor for endometrial adenocarcinoma. Clin Transl Oncol. (2015) 17:835–9. doi: 10.1007/s12094-015-1309-8 26260911

[20] Jin J Dalwadi SM Masand RP Hall TR Anderson ML Ludwig MS . Association between metabolic syndrome and endometrial cancer survival in a SEER-medicare linked database. Am J Clin Oncol. (2020) 43:411–7. doi: 10.1097/COC.0000000000000686 32205571

[21] Kokts-Porietis RL McNeil J Nelson G Courneya KS Cook LS Friedenreich CM . Prospective cohort study of metabolic syndrome and endometrial cancer survival. Gynecol Oncol. (2020) 158:727–33. doi: 10.1016/j.ygyno.2020.06.488 32600790

[22] Shou H Yan K Song J Zhao L Zhang Y Ni J . Metabolic syndrome affects the long-term survival of patients with non-endometrioid carcinoma of the uterine corpus. Int J Gynaecol Obstet. (2020) 148:96–101. doi: 10.1002/ijgo.v148.1 31560127

[23] Shafiee MN Razak N Ahmad MF Abd Aziz N Adeeb N . A single centre experience of metabolic syndrome and endometrial carcinoma: 5 years review. J Obstet Gynaecol. (2021) 41:285–9. doi: 10.1080/01443615.2020.1819210 33258710

[24] Yang X Li X Dong Y Fan Y Cheng Y Zhai L . Effects of metabolic syndrome and its components on the prognosis of endometrial cancer. Front Endocrinol (Lausanne). (2021) 12:780769. doi: 10.3389/fendo.2021.780769 34975754 PMC8717682

[25] Chen MQ Lin HX Liang JX Wu MF Li J Wang LJ . Association between subtypes of metabolic syndrome and prognosis in patients with stage I endometrioid adenocarcinoma: A retrospective cohort study. Front Oncol. (2022) 12:950589. doi: 10.3389/fonc.2022.950589 36203442 PMC9530564

[26] Wang Z Song K Liu J Zhang Q Zhang C Wang B . Prognostic-related metabolic score for survival prediction in early-stage endometrioid endometrial cancer: A multi-center and retrospective study. Front Med (Lausanne). (2022) 9:830673. doi: 10.3389/fmed.2022.830673 35573009 PMC9096267

[27] Shehaj I Krajnak S Rad MT Gasimli B Hasenburg A Karn T . Prognostic impact of metabolic syndrome in patients with primary endometrial cancer: a retrospective bicentric study. J Cancer Res Clin Oncol. (2024) 150:174. doi: 10.1007/s00432-024-05699-1 38570343 PMC10991018

[28] Page MJ McKenzie JE Bossuyt PM Boutron I Hoffmann TC Mulrow CD . The PRISMA 2020 statement: an updated guideline for reporting systematic reviews. BMJ. (2021) 372:n71. doi: 10.1136/bmj.n71\ 33782057 PMC8005924

[29] Page MJ Moher D Bossuyt PM Boutron I Hoffmann TC Mulrow CD . PRISMA 2020 explanation and elaboration: updated guidance and exemplars for reporting systematic reviews. BMJ. (2021) 372:n160. doi: 10.1136/bmj.n160 33781993 PMC8005925

[30] Higgins J Thomas J Chandler J Cumpston M Li T Page M . Cochrane Handbook for Systematic Reviews of Interventions version 6.2. London, UK: The Cochrane Collaboration (2021). Available at: www.training.cochrane.org/handbook.

[31] Wells GA Shea B O'Connell D Peterson J Welch V Losos M . The Newcastle-Ottawa Scale (NOS) for Assessing the Quality of Nonrandomised Studies in Meta-Analyses (2010). Available online at: http://www.ohri.ca/programs/clinical_epidemiology/oxford.asp (Accessed May 3, 2021).

[32] Higgins JP Thompson SG . Quantifying heterogeneity in a meta-analysis. Stat Med. (2002) 21:1539–58. doi: 10.1002/sim.v21:11 12111919

[33] Egger M Davey Smith G Schneider M Minder C . Bias in meta-analysis detected by a simple, graphical test. BMJ. (1997) 315:629–34. doi: 10.1136/bmj.315.7109.629 PMC2127453 9310563

[34] Karra P Winn M Pauleck S Bulsiewicz-Jacobsen A Peterson L Coletta A . Metabolic dysfunction and obesity-related cancer: Beyond obesity and metabolic syndrome. Obes (Silver Spring). (2022) 30:1323–34. doi: 10.1002/oby.23444 PMC9302704 35785479

[35] Majchrzak-Baczmanska D Malinowski A . Does IGF-1 play a role in the biology of endometrial cancer? Ginekol Pol. (2016) 87:598–604. doi: 10.5603/GP.2016.0052 27629137

[36] Bruchim I Sarfstein R Werner H . The IGF hormonal network in endometrial cancer: functions, regulation, and targeting approaches. Front Endocrinol (Lausanne). (2014) 5:76. doi: 10.3389/fendo.2014.00076 24904527 PMC4032924

[37] Tumminia A Vinciguerra F Parisi M Graziano M Sciacca L Baratta R . Adipose tissue, obesity and adiponectin: role in endocrine cancer risk. Int J Mol Sci. (2019) 20(12):2863. doi: 10.3390/ijms20122863 31212761 PMC6628240

[38] Li R Dong F Zhang L Ni X Lin G . Role of adipocytokines in endometrial cancer progression. Front Pharmacol. (2022) 13:1090227. doi: 10.3389/fphar.2022.1090227 36578551 PMC9791063

[39] Bacalbasa N Diaconu C Iliescu L Savu C Balalau C Dimitriu M . The influence of the metabolic syndrome on early postoperative outcomes of patients with advanced-stage endometrial cancer. In Vivo. (2020) 34:2913–7. doi: 10.21873/invivo.12120 PMC7652503 32871832

[40] Qiang Y Zhang Q Dong L . Metabolic risk score as a predictor in a nomogram for assessing myometrial invasion for endometrial cancer. Oncol Lett. (2023) 25:114. doi: 10.3892/ol.2023.13700 36844632 PMC9950329

